# Kinase Inhibitors from Marine Sponges

**DOI:** 10.3390/md9102131

**Published:** 2011-10-24

**Authors:** Danielle Skropeta, Natalie Pastro, Ana Zivanovic

**Affiliations:** 1 School of Chemistry, University of Wollongong, Wollongong, NSW 2522, Australia; E-Mails: natalie.pastro@gmail.com (N.P.); az993@uowmail.edu.au (A.Z.); 2 Centre for Medicinal Chemistry, University of Wollongong, Wollongong, NSW 2522, Australia

**Keywords:** marine sponges, marine natural products, protein kinase inhibitors, cell regulation, anticancer agents

## Abstract

Protein kinases play a critical role in cell regulation and their deregulation is a contributing factor in an increasing list of diseases including cancer. Marine sponges have yielded over 70 novel compounds to date that exhibit significant inhibitory activity towards a range of protein kinases. These compounds, which belong to diverse structural classes, are reviewed herein, and ordered based upon the kinase that they inhibit. Relevant synthetic studies on the marine natural product kinase inhibitors have also been included.

## 1. Introduction

The search for pharmaceutically active compounds from natural sources is well established, with approximately 70% of small molecule drugs produced between 1981 and 2006 possessing an important link to a natural product source [1]. The pharmaceutical value of natural products is even more exemplified in the critical area of anticancer drugs, whereupon of the 155 small-molecules produced from the 1940s, 73% are other than “synthetic”, with 47% being natural products or natural product derived [1]. With oceans covering 70% of the surface of the earth, coupled with the large and varied biodiversity of the marine environment, the oceans remain a largely unexplored, but extremely promising source of new drug candidates. Approximately half of the novel marine natural products reported in the literature are biologically active [2]. This occurrence can be contributed to the reliance of sessile, soft-bodied marine invertebrates on chemical defense for survival, as many lack the physical defense mechanisms of movement and camouflage. As these chemicals are released into the water and are rapidly diluted, these secondary metabolites produced are often extremely potent [3].

The protein kinase family encompasses all enzymes in the human body that catalyse the chemical transfer of a phosphate group from a high energy molecule such as adenine triphosphate (ATP) to a specific substrate. The human genome encodes for approximately 518 different protein kinases, which are divided into different kinase families on the basis of their selectivity for substrates [4]. The covalent attachment of a phosphate group to a substrate requires a free hydroxyl moiety, and there are three amino acids that can provide this; serine, threonine and tyrosine. Therefore, serine/threonine kinases will recognise and attach a phosphate group to a serine or threonine amino acid, while the tyrosine-specific protein kinase family will phosphorylate a protein at a tyrosine moiety.

Kinases play a large, varied and vital role in cell regulation and particularly in signal transmission pathways, controlling cell differentiation, proliferation, metabolism, DNA damage repair, cell motility, response to external stimuli and apoptosis [5]. Deregulation of kinases has been found to be a primary cause in an increasing list of diseases, including oncological diseases, central nervous system disorders, autoimmune diseases, metabolic diseases and osteoporosis, suggesting that the number of kinases with the potential to be new pharmaceutical targets is significantly large [6]. The current focus on kinases is in the development of drugs with lower side effects than previous cancer treatments which traditionally focused on DNA replication and chromosome regulation and thus also affected many healthy cells. As various kinases have been reported to be misregulated in cancerous cells, anticancer treatments involving kinases can be specifically targeted to cancer cells [4]. The development of kinase inhibitors has been predicted to be a major driver of pharmaceutical growth with more than 130 kinase inhibitors reported to be in either Phase I or Phase II clinical trials, the majority of these being tested for their potential as cancer treatments [7]. Kinase inhibitors that successfully proceed onto the pharmaceutical market will join Imitinib (Gleevec, Novartis), a tyrosine kinase inhibitor that has dramatically improved the prognosis for sufferers of chronic myeloid leukemia after being the first small-molecule kinase inhibitor to be approved for human use [6]. Herein, we review the recent highlights and developments of over 70 kinase inhibitors that have been isolated from marine sponges.

## 2. Reviews

Kinase inhibitors and activators from natural sources were covered in 2011 by Marston in a review that included a small number of marine natural products [8]. In 2007, Nakao and Fusetani published a review on enzyme inhibitors isolated from marine organisms which included some protein kinase inhibitors from marine sponges [9]. In 2009, Deslandes *et al.* reviewed the synthesis and kinase inhibitory activities of the marine natural products granulatimide and isogranulatimide [10]. In 2009, Nguyen *et al.* published the synthesis and evaluation of the kinase inhibitory activity of the sponge derived compound hymenialdisine and its analogues [11]. In 1998, Carter and Kane reviewed the therapeutic potential of natural compounds that regulate the activity of protein kinase C [12]. To the best of the authors’ knowledge, this is the first comprehensive review that is focussed solely on the kinase inhibitory activities of marine sponge metabolites.

## 3. Protein Kinase C (PKC, EC 2.7.11.13)

The family of kinases known as protein kinase C (PKC) are serine/threonine kinases that encompass eleven isozymes and through the action of phosphorylating various intracellular proteins, mediate many physiological events such as induction of cell differentiation, regulation of apoptosis and inhibition of tumor invasion [13]. Protein kinase C is composed of two distinct regions; a carboxyl-terminal catalytic site containing an adenine triphosphate (ATP) binding site and a regulatory domain at the amino terminal that possesses a phorbol-binding domain that is unique to the PKC family [14]. The catalytic site on PKC is structurally shared amongst many different classes of kinases, and as such PKC inhibitors that block this site can also inhibit the action of other functionally diverse kinases [14]. Natural activators of PKC include diacylglycerols, phosphatidyl serine, inositol triphosphate and calcium ions. The vital role that PKCs play in signal transduction pathways has marked them as potential targets for pharmaceutical inhibition of diseases such as cancer, cardiovascular disease, renal disease, immunosuppression and autoimmune disease [15].

The efficacy of the natural product staurosporine as a PKC inhibitor has been known since last century when the alkaloid was isolated from the bacteria *Streptomyces staurosporeus* and shown more recently to have an IC_50_ value of 2.7 nM against PKC [16]. In recent years, a variety of marine organisms have also provided important PKC modulators such as 11-hydroxystaurosporine from the marine tunicate *Eudistoma* sp. [17] and bryostatin-1, from the marine bryozoan *Bugula neritina* [14,18]. Marine sponges have also proven to be a particularly rich source of PKC inhibitors.

**Figure 1:**
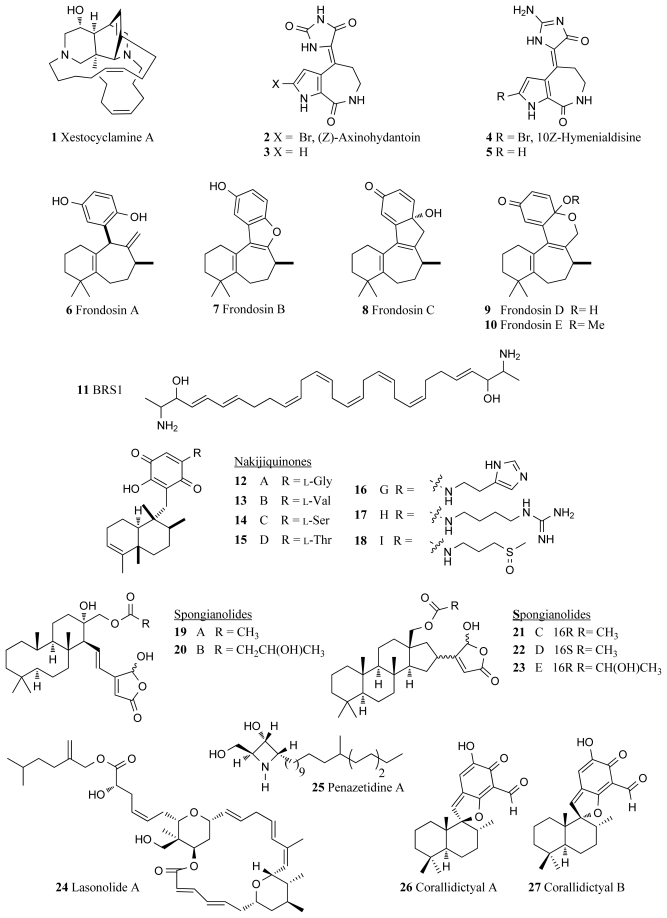
Protein kinase C inhibitors isolated from marine sponges.

In 1994, the sponge *Xestospongia* sp. collected in waters off the Papua New Guinea coast, furnished xestocyclamine A (**1**, Figure 1) bearing a novel skeleton and found to inhibit PKC with an IC_50_ value of 4 μg/mL [19]. Xestocyclamine A and its pure enantiomer (−)-xestocyclamine A are considered critical PKC inhibitors for use in the development of anticancer drugs and there are many research groups focused on synthesising the stereochemically complex marine alkaloids [20,21]. (*Z*)-Axinohydantoin (**2**, Figure 1) and debromo-*Z*-axinohydantoin (**3**, Figure 1) are two PKC inhibitors with respective IC_50_ values of 9.0 and 22.0 μM that were isolated from the marine sponge *Stylotella aurantium* [22]. These novel compounds were isolated during a scale-up collection of the PKC inhibitors, hymenialdisine (**4**, IC_50_ 0.8 μM, Figure 1) and debromohymenialdisine (**5**, IC_50_ 1.3 μM, Figure 1) from the same sponge species [22]. Hymenialdisine is found to inhibit a range of kinases (see Section 4.1).

Five novel sesquiterpene derivatives, frondosins A–E (**6**–**10**, Figure 1), were isolated from the marine sponge *Dysidea frondosa* and shown to have inhibitory activity against PKC with reported IC_50_ values of 1.8, 4.8, 20.9, 26.0 and 30.6 μM respectively [23]. Frondosins A–E were also reported to be inhibitors of interleukin-8 in the low micromolar range [23] and more recently (−)-frondosins A (**6**) and D (**9**) have shown comparable activity against the HIV virus [24]. Various synthetic routes to frondosins A–C have been reported [25–27].

BRS1 (**11**, Figure 1), a polyunsaturated lipid isolated from an unidentified Australian sponge of class *Calcarea* was reported to be a novel inhibitor of PKC [28]. BRS1 exerts it activity by binding to the phorbol ester binding site and accounts for 0.02% of the wet weight of the sponge from which it was collected. The IC_50_ of BRS1 for inhibiting the binding of the phorbol ester was 9 μM, whereas 98 μM represented a 50% effective concentration for inhibiting the enzymatic activity of PKC [28].

An Okinawan marine sponge belonging to the family Spongiidae, has furnished a family of novel sesquiterpenoid quinones, including the nakijiquinones A–D (**12**–**15**, Figure 1), with reported IC_50_ values against PKC of 270, 200, 23 and 220 μM respectively [29,30]. A subsequent paper described the isolation of the nakijiquinones G–I (**16**–**18**) from the same sponge, which showed modest cytotoxicity in the range of 2.4 to >10 μg/mL against a range of cancer cell lines (e.g., P388 murine leukemia, L1210 murine leukemia and KB human epidermal carcinoma cells), as well as inhibitory activity against HER2 kinase [31]. The remarkable inhibitory activity of nakijiquinones A–D against a variety of kinases including epidermal growth factor receptor (EGFR), c-erbB-2 kinase and tyrosine kinase VEGFR2 has been reviewed and their biological activity and structure-activity relationships are well documented [15,32]. The synthesis of the nakijiquinones has been reported [33,34], with particular emphasis on the potential of nakijiquinone and its analogues in the prevention of angiogenesis as the nakijiquinone family is the only naturally occurring inhibitor of the Her-2/Neu receptor tyrosine kinase. Extensively implicated in tumor proliferation, the Her-2/Neu receptor tyrosine kinase is over-expressed in approximately 30% of primary breast, ovary and gastric cancers and when amplified has been linked to increases in the aggressiveness of the cancer and reduced patient survival [35].

The cytotoxic sesterpenes spongianolides A–E (**19**–**23**, Figure 1), isolated from a marine sponge belonging to the genus *Spongia*, were found to have inhibitory activity against PKC with IC_50_ values ranging between 20–30 μM [36]. The cheilanthane cyclic terpenoid contained within the structure has since been synthesized via a biomimetic approach [37] (see also Section 7).

Another potent marine sponge derived PKC inhibitor is lasonolide A (**24**, Figure 1). Isolated from the Caribbean sponge *Forcepia* sp., lasonolide A was found to inhibit the phorbol ester-stimulated adherence of EL-4.IL-2 mouse thymoma cells within 30 min with an IC_50_ value of 27 nM, highlighting the potential of this compound for development as a potent PKC inhibitor [38–40].

Another inhibitor of PKC enzyme is a new azetidine compound penazetidine A (**25**, Figure 1) isolated from the Indo-Pacific marine sponge *Penares sollasi* [41]. This sponge species attracted attention after its crude extract in initial screenings exhibited inhibitory activity (IC_50_ 0.3 μg/mL) against serine kinase PKC-βI, but it was not active against protein tyrosine kinase (PTK). Penazetidine A displayed strong activity against PKC (IC_50_ l μM), and also showed significant cytotoxicity against human and murine cancer cell lines (A549, HT-29, B16/F10 and P388) [41]. A mixture of two diastereomeric spirosesquiterpene aldehydes, corallidictyals A and B, were isolated from the marine sponge *Aka* (*=Siphonodictyon) coralliphaga*, which was collected at Little San Salvador Island (**26**, **27**, Figure 1) [42]. This mixture was found to show good selectivity for the inhibition of PKC (IC_50_ 28 μM) compared to the other serine kinase enzyme PKA (IC_50_ 300 μM). In particular, the diastereomeric mixture was selective for inhibition of the α-PKC isoform giving a lower IC_50_ value compared to the other isoforms of the enzyme.

## 4. Cyclin Dependent Kinases (CDK, EC 2.7.11.22)

Cyclin-Dependent kinases (CDKs) are a group of serine/threonine kinases that encompass approximately 25 different cyclin families, all of which are critical in the regulation of the cell cycle [4]. The distinguishing feature of the CDKs from other kinase families is the enzymatic activation requirement of the binding of the cyclin regulatory subunit [43]. The movement of the cell through the cell cycle phases is determined by the fluctuating concentrations of different activated CDK/cyclin complexes whose cellular mechanism involves the phosphorylation of many distinct proteins at serine or threonine residues in specific sequences. While CDKs are also involved in apoptosis and transcription, their pivotal role in differentiation, transformation, proliferation and metastasis has recently seen CDKs become a major target for cancer therapies, especially now that it is recognized that hyperactive CDKs (overexpression) or hypoactive CDKs (mutation, deletion) are a leading cause of uncontrolled tumor proliferation in humans [4]. Several natural and synthetic compounds that inhibit CDKs in the sub-micromolar range have been isolated and are at various stages of clinical trials, the most advanced being flavopiridol, a semi-synthetically produced analogue of an alkaloid from the Indian tree *Dyoxylum binectariferum*, currently in Phase II clinical trials for soft tissue sarcomas [44]. These small molecule inhibitors arrest tumor proliferation and many are also capable of inducing apoptosis in proliferating cells [45].

### 4.1. Cyclin Dependent Kinase-1

Cyclin Dependent Kinase-1 (CDK-1) is a critical controller of the cell cycle in multi-cellular eukaryotic organisms and operates primarily in the mitosis (M) phase. In order for the cell to pass from the growth (G2) phase into M phase, activation of the CDK-1/cyclin B1 complex must be sustained in the nucleus from prophase into metaphase [46]. Hymenialdisine (**4**, Figure 1), a potent inhibitor of CDK-1, was first isolated in 1982 from the marine sponges *Axinella verrucosa* and *Acanthella aurantiaca* [47]. Hymenialdisine inhibits CDK-1/cyclin B (IC_50_ 22 nM) through competitive inhibition at the ATP-binding site, and as this site is homologous with many kinase families, hymenialdisine also shows inhibitory activity against a variety of different kinases including CDK-2/cyclin A (IC_50_ 70 nM), CDK-2/cyclin E (IC_50_ 40 nM), CDK-5/p25 (IC_50_ 28 nM), glycogen synthase kinase 3 (GSK-3) (IC_50_ 10 nM) and creatine kinase 1 (CK1) (IC_50_ 35 nM), while still possessing good selectivity *in vitro* as inhibition of alternate molecular targets occurs at much higher IC_50_ values [48].

**Figure 2:**
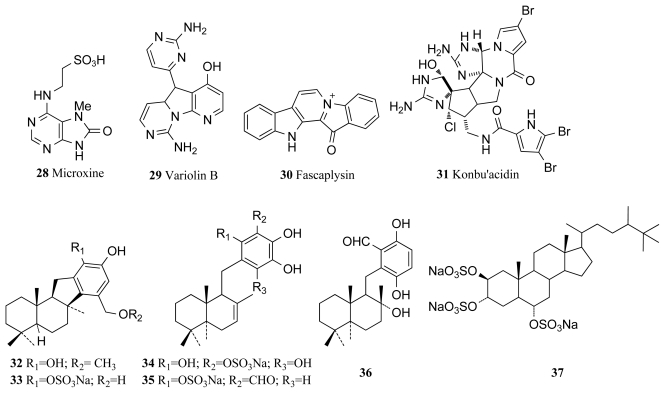
Cyclin dependant kinase inhibitors isolated from marine sponges.

Inhibition of the CDK-1/cyclin B complex has recently been shown to induce apoptosis in cells experiencing Myc (proto-oncogene) overexpression [49], a common phenomenon in many human cancers and a mechanism by which hymenialdisine and associated analogues could potentially act as anticancer agents. Many analogues of hymenialdisine that exhibit inhibitory activity against various CDKs in the nanomolar range have been successfully synthesized as medicinal chemists recognised the potential of hymenialdisine for use against many degenerative diseases [50]. Recent patents also highlight hymenialdisine and analogues as likely future pharmaceuticals for diseases such as asthma, rheumatoid arthritis, multiple sclerosis and Alzheimer’s disease due to its ability to arrest the NF-kappa B signaling process, a critical mechanism in the above diseases [51]. Microxine (**28**, Figure 2), a novel purine derivative, is an inhibitor of CDK-1, isolated from the Australian marine sponge genus *Microxina*, with an IC_50_ value of 13 μM against CDK-1 [52,53]. Variolin B (**29**, Figure 2) was isolated from the Antarctic sponge *Kirkpatrickia varialosa*, and it was found to display CDK inhibitory activity exhibiting selective inhibition towards CDK-1 and CDK-2 over CDK-4 and CDK-7 [53,54]. It was hypothesized that mechanism of action of variolin B is the inhibition of cyclin-dependent kinases that interrupt the progression of the normal cell cycle. Variolin B inhibits the phosphorylation of histone H1 mediated by CDK-2/cyclin E, CDK-2/cyclin A, CDK-1/cyclin B, CDK-7/cyclin H, and CDK4/cyclin D, with IC_50_ values in the micromolar range [53]. Total synthesis of this compound has been performed by several research groups due to the vast biological potential of the compound with its antiviral and antitumor activity, including cytotoxicity towards the P388 murine leukemia cell line with an IC_50_ value of 210 ng/mL [53,55–58].

### 4.2. Cyclin Dependent Kinase-4

Another member of the CDK family is CDK-4, a catalytic subunit whose presence is vital for the progression of the cell cycle through the G1 phase [59]. The activity of CDK-4 is restricted to the G1-S phases and is regulated by the attachment of the regulatory subunit cyclin D and the endogenous CDK inhibitor p16(INK4a). The G1-S checkpoint is the most important regulation point in the cell cycle, exemplified by the fact that the G1-S transition is misregulated in 60–70% of cancers [60]. A major role of CDK-4 is the phosphorylation of the retinoblastoma gene product (Rb) [59]. A high incidence of mutations in Rb, along with cyclin D and p16(INK4a), has been seen in tumorigenesis in many cancers, a fact which has recently seen CDK-4 become an exciting new cancer drug target.

A major distinguishing feature of fascaplysin (**30**, Figure 2), a red pigment isolated from the marine sponge *Fascaplysinopsis* sp. is that it is a selective inhibitor of CDK-4 [61]. Poor selectivity is a common problem among kinase inhibitors due to the ATP binding site, where many inhibitors exert their actions, being conserved amongst the majority of kinase families. Fascaplysin exhibits an IC_50_ value of 0.35 μM against the CDK-4/cyclin D complex while IC_50_ values against other kinases were comparably much higher [61]. This specificity allows fascaplysin to be a useful scientific tool in investigating the direct consequences of singular CDK-4 inhibition [61] and many studies have thus been conducted establishing the potential of fascaplysin as a pharmaceutical agent. A recent study has identified fascaplysin as a natural angiogenesis inhibitor after it was found that fascaplysin selectively inhibited the proliferation of endothelial cells toward tumor cells and suppressed the vascular endothelial growth factor (VEGF), a critical player in angiogenesis [60]. Conclusions from such studies indicate that fascaplysin could in the future play a central role in preventing cancers from metastasizing and becoming malignant by preventing new vascular growth at the tumor site [60].

Konbu’acidin A (**31**, Figure 2) is a novel bromopyrrole alkaloid that was isolated from the Okinawan marine sponge *Hymeniacidon* sp. and reported to display inhibitory activity against the CDK-4/cyclin D complex [62]. Konbu’acidin A showed inhibitory activity against CDK-4 with an IC_50_ of 20 μg/mL but did not show any cytotoxicity against murine leukemia L1210 and epidermal carcinoma KB cell lines [62]. The marine sponge *Aka* sp. collected from Micronesia yielded three novel sesquiterpene quinols (**32**–**34**), two known quinols (**35**, **36**) and halistanol sulfate (**37**). Four of the compounds (**32**, **35**–**37**) were screened for CDK/cyclin D1 kinase inhibitory activity and compounds **35** and **37** exhibited moderate kinase inhibitory activity and inhibited complex formation with IC_50_ values of 9.0 and 9.5 μg/mL respectively [63].

## 5. Tyrosine Protein Kinase (TPK, EC 2.7.10.1)

Tyrosine protein kinase (TPK) are enzymes that catalyse the phosphorylation of tyrosine residues and can be divided into two main categories; cellular and receptor TPKs, and non-receptor TPKs. Studies into this particular class of kinase have identified them as key players in both intracellular and extracellular communication [64]. TPKs are associated with proliferative diseases such as cancer, leukemia, psoriasis and restonosis due to their role in regulating key cell functions like proliferation, differentiation, and antiapoptotic signaling [64] and it has been reported that 70% of the known oncogenes and proto-oncogenes found in cancer are associated with TPKs [65].

**Figure 3:**
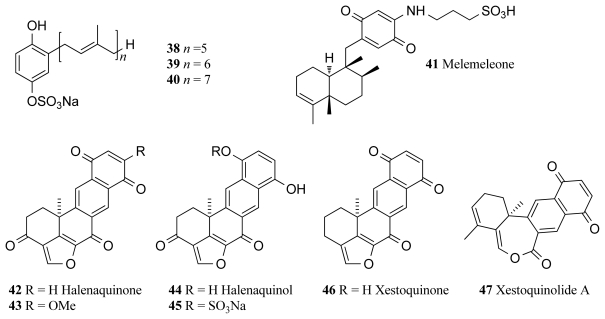
Tyrosine protein kinase inhibitors isolated from marine sponges.

The deep-sea sponge *Ircinia* sp. collected off the New Caledonian coast at a depth of 425–500 m yielded three TPK inhibitors, the penta-, hexa- and hepta-prenylhydroquinone 4-sulfates (**38**–**40**, Figure 3). IC_50_ values for each compound against TPK were recorded as 8, 4 and 8 μg/mL respectively [66]. Penta-prenylhydroquinone sulfate (**38**, Figure 3) has also proven to be a potential antiviral and cytotoxic agent achieving 65% inhibition of the HIV-1 integrase enzyme at 1 μg/mL and having inhibited neuropeptide Y (NPY) receptor with an IC_50_ value of 50.8 μg/mL. This compound also displayed cytotoxicity against the epidermal KB carcinoma cell line [66].

### Tyrosine Kinase pp60^V-SRC^

Tyrosine kinase pp60^V-SRC^ is a membrane-associated protein with protein kinase activity and is also the oncogene product of the Rous Sarcoma retrovirus, which upon entry into a cell, transforms a normal cell into a rapidly proliferating cell [67]. Melemeleone (**41**, Figure 3) is a novel sesquiterpene quinonecompound, isolated along with another four new metabolites and two known compounds, from two sponge species of *Dysidea* from Solomon Island [68]. All purified compounds isolated from the sponge were tested for kinase inhibitory activity, but only melemeleone displayed activity against pp60^V-SRC^ with an IC_50_ of 28 μM [68].

Several inhibitors of pp60^V-SRC^ were isolated from the Fijian sponge *Xestospongia carbonaria*, namely halenaquinone (**42**, Figure 3), halenaquinol (**44**, Figure 3), halenaquinol sulfate (**45**, Figure 3) and xestoquinone (**46**, Figure 3), and reported IC_50_ values of 1.5, 60.0, 0.55 and 28.0 μM, respectively [69]. Of these pentacyclic polyketide compounds, halenaquinone proved to be the most pharmaceutically promising, due to its characterisation as an irreversible inhibitor. The potential of halenaquinone as an anticancer agent is evidenced by findings that it arrests the proliferation of various cell lines, including those that have been transformed by oncogenic PTKs, and halenaquinone also shows inhibitory activity against the kinase activity of the human EGFR with an IC_50_ value of 19 μM [69]. Halenaquinone (**42**) and xestoquinone (**46**) were also isolated from the same sponge *Xestospongia* sp. collected from Vanuatu and were found to inhibit several kinases. Xestoquinone inhibited Pfnek-1 kinase of *Plasmodium falciparum* with IC_50_ of 1.1 μM but displayed lower kinase inhibitory activity towards PfPK5 and no activity towards PfPK7 and PfGSK-3 [70].

Halenaquinone and halenaquinol have since been associated with antibiotic and cardiotonic activity in addition to their ability to inhibit pp60^V-SRC^, and have been the focus of several synthetic studies [71]. Strategies for the synthesis of the core skeletons of halenaquinone and halenaquinol have recently been described with the construction of the furan-fused tetracyclic core of the molecules. The key step involved the intramolecular [4 + 2]-cycloaddition reaction of *o*-quinodimethane [71]. The current highlight with halenaquinone is as its potential as an inhibitor of recombinant human Cdc25B phosphatase [72], an activator of cyclin dependent kinase Cdc2 whose presence is required for entry into the mitosis phase of the cell cycle. Displaying an IC_50_ value of 0.7 μM, halenaquinone stands out as a key molecule in anticancer studies revolving around this drug target [72]. Alvi *et al.* also isolated the two compounds 14-methoxyhalenaquinone (**43**, Figure 3) and xestoquinolide A (**47**, Figure 3) from the same sponge, for which IC_50_ values of 5 and 80 μM against protein tyrosine kinase (PTK) respectively were reported [73].

## 6. Epidermal Growth Factor Receptor (EC 2.7.10.1)

**Figure 4:**
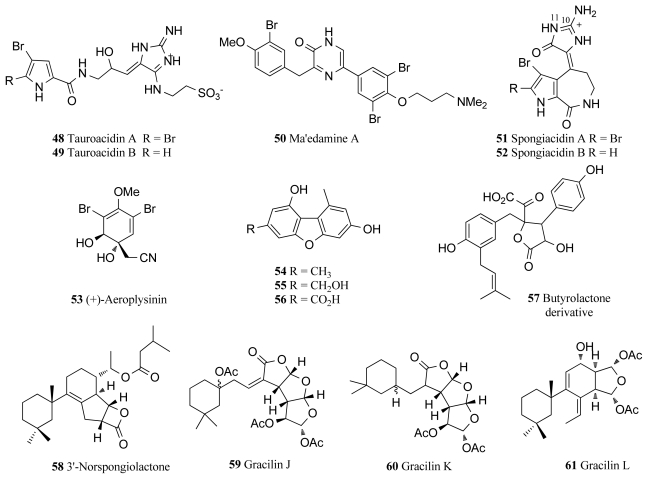
Epidermal growth factor receptor inhibitors isolated from marine sponges.

The epidermal growth factor receptor (EGFR) is a member of the type 1 growth factor receptor gene family, which also includes erbB-1, erbB-2, erbB-3 and erbB-4 [74]. This tyrosine kinase family has been heavily implicated in the mechanisms of various cancers as mutations leading to EGFR being over-expressed have often been found in cancer cases, in particular breast cancer [15]. As part of an extensive effort to identify small molecule inhibitors of this drug target, two novel bromopyrrole alkaloids were isolated from an Okinawan marine sponge *Hymeniacidon* sp. and named tauroacidins A and B (**48**, **49**, Figure 4) [75]. These two compounds showed inhibitory activity against both EGFR and c-erbB-2 kinase with an IC_50_ value of 20 μg/mL for each respective kinase [75]. The tauroacidins A and B may be biogenetically related to other bromopyrrole alkaloids from marine sponges through the taurine residue attached to the aminoimidazole ring [75]. Okinawan marine sponges have proven to be a particularly rich source of kinase inhibitors with a bromotyrosine alkaloid, ma’edamine A (**50**, Figure 4), also being isolated from the Okinawan marine sponge *Suberea* sp. and showing inhibitory activity against c-erbB-2 kinase (IC_50_ 6.7 μg/mL) [76]. Ma’edamine A contains a unique 2(1*H*)pyrazinone moiety located between the two bromotyrosine units, and also displays cytotoxicity against murine leukemia L1210 cells (IC_50_ 4.3 μg/mL) and epidermal KB carcinoma cells (IC_50_ 5.2 μg/mL) [76].

Spongiacidins A and B (**51**, **52**, Figure 4) are inhibitors of c-erbB-2 kinase isolated from the Okinawan marine sponge *Hymenacidon* sp. [77]. These two compounds are also bromopyrrole alkaloids of the pyrrolo[2,3-*c*]azepine type. The respective IC_50_ values for spongiacidins A and B against c-erbB-2 kinase are 8.5 and 6.0 μg/mL [77]. It was later identified that spongiacidin A is actually the (*E*) isomer at the exocyclic C10-C11 double bond of 3-bromohymenialdisine, a metabolite of hymenialdisine discussed earlier [78].

Isolated from the marine sponge species *Verongia aerophoba*, (+)-aeroplysinin-1 (**53**, Figure 4) was found to completely inhibit EGFR at a concentration of 0.5 μM [79]. Due to this inhibitory ability, (+)-aeroplysinin-1 was found to have a strong antitumor effect on EGFR tumor cell lines, in particular blocking the proliferation of EGFR dependent human breast cancer cell lines MCF-7 and ZR-75-1 [79]. Importantly, (+)-aeroplysinin-1 displays some selectivity for cancerous cells as the application of (+)-aeroplysinin-1 at a concentration of 0.25–0.5 μM resulted in total tumor cell death, but did not have any cytotoxic effect on normal human fibroblasts at concentrations ten times higher [79]. A recent study has identified (+)-aeroplysinin-1 as an important inhibitor of several key steps of angiogenesis, the process by which tumors become mutagenic and thus a vital target for pharmaceutical intervention in cancerous diseases [80]. In detail, (+)-aeroplysinin-1 has been shown to inhibit capillary-like tube formation, induce apoptosis, promote anti-proteolysis in endothelial cells and also arrest the development of new vascular structures [80]. As angiogenesis is a major factor in fatal cancers and (+)-aeroplysinin-1 displays *in vivo* efficacy as an inhibitor of this process, it remains an extremely promising drug candidate.

Three novel compounds identified as 3,9-dimethyldibenzo[*b*,*d*]furan-1,7-diol (**54**), 3-(hydroxymethyl)-9-methyldibenzo[*b*,*d*]furan-1,7-diol (**55**), 1,7-dihydroxy-9-methyldibenzo[*b*,*d*] furan-3-carboxylic acid (**56**) and one known compound, butyrolactone derivative (**57**), were isolated from marine sponge *Acanthella cavernosa* from Fiji and all compounds displayed moderate inhibitory properties against EGFR [81]. In recent studies, bioassay-guided fractionation of the marine sponge *Spongionella* sp., yielded the novel bioactive diterpenes, 3′-norspongiolactone (**58**, Figure 4) and gracilins J–L (**59**–**61**), along with three known gracilins and the known diterpenoid tetrahydroaplysulphurin-1 [82]. All eight compounds isolated from the sponge *Spongionella* sp. exhibited cytotoxicity against the K562 human chronic myelogenous leukemia cell lines with IC_50_ values in the range of 0.6 to 15 μM, however they also showed similar levels of cytotoxicity towards human peripheral blood mononuclear cells (PBMC) [82]. All compounds displayed inhibitory activity towards EGFR tyrosine kinase with the novel diterpenes **58**–**61** exhibiting 25%, 19%, 75% and 57% inhibition respectively at 100 μM [82].

## 7. Mitogen-Activated Protein Kinase (EC 2.7.11.24)

Mitogen- and stress-activated kinase (MSK1) and mitogen-activated protein kinase (MAPK) are two stress-associated serine/threonine specific protein kinases involved in cellular signaling, regulating various processes such as cell division and proliferation, apoptosis and gene expression [83]. There are three major subclasses of this kinase family, including extracellular signal-regulated kinases (ERKs), c-Jun *N*-terminal kinase (JNK)/stress-activated protein kinase (SAPKs) and p38 MAPKs [83]. It has been acknowledged that selective inhibitors of these kinases are likely to affect cellular events with high specificity and are therefore molecules of significant interest in the search for anticancer pharmaceuticals [15].

**Figure 5:**
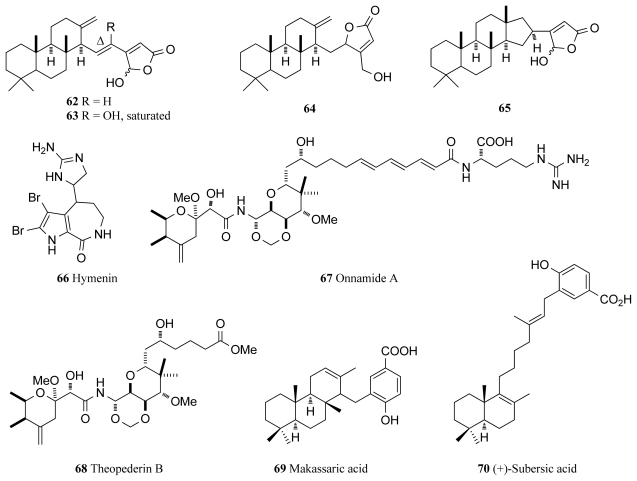
Mitogen-activated protein kinase inhibitors isolated from marine sponges.

In the first description of cheilanthane sesterterpenoids from a marine sponge, three novel (**62**–**64**) and one known cheilanthane sesterterpenoids (**65**, Figure 5) were isolated from the marine sponge *Ircinia* sp., with **62**, **63** and **65** obtained as inseparable 1:1 mixtures of their C-25 epimers. Intriguingly, all four compounds were reported to exhibit identical inhibitory activity against MSK1 (IC_50_ 4 μM for each compound) and mitogen activated protein kinase activated protein kinase (MAPKAPK-2, IC_50_ 90 μM for each compound) [84]. Extracts from two sponge species, the purple bleeding sponge *Iotrochota birotulata* and the West Indian bath sponge *Spongia barbara* were found to inhibit the MAPK/ERK cascade, a pathway that links the binding of growth factors on cell surface receptors to intracellular responses [85]. Encompassing many protein kinases, activation of this cascade leads to cell division and is thus a potential anticancer drug target [86]. The two extracts significantly inhibited the MAPK/ERK pathway to 51% and 44% of control levels respectively without affecting the survival of the cell [85].

### Raf (EC 2.7.11.1)/MAP Kinase Kinase (EC 2.7.12.2)/MAPK (EC 2.7.11.24)

The Raf kinase, MAP kinase kinase (MEK) and MAPK combine to form a pathway that links extracellular signals to the phosphorylation of cellular proteins to regulate cell proliferation and differentiation [87]. The cascade is firstly activated by Ras promoting the translocation of Raf-1 to the inner cell membrane where it undergoes phosphorylation for activation. Raf-1 specifically phosphorylates and activates MEK, which will continue the process by phosphorylating MAPKs, causing them to migrate into the nucleus of the cell and influence many cellular events [87]. The oncogenic form of Ras is implicated in over 30% of all cancers, and as the Raf/MEK/MAPK cascade contains many potential sites for inhibition, this is an important and extremely promising target to be studied for pharmaceutical intervention [87].

While it has been known for some time that hymenialdisine (**4**, Figure 1) shows significant inhibitory activity against many cellular kinases, it has recently been reported that hymenialdisine and debromohymenialdisine (**5**, Figure 1) are remarkably potent inhibitors of MEK with IC_50_ values of 3.0 and 6.0 nM respectively [87]. These two compounds, isolated from the marine sponge *Stylotella aurantium* arrest the Raf/MEK/MAPK cascade by specifically binding to and inhibiting the phosphorylation of MAPK by MEK-1. It is also believed that 10*E*-hymenialdisine spontaneously converts to 10*Z*-hymenialdisine (**4**, Figure 1) on standing [87] and the mixture of these two compounds was shown to have the ability to inhibit the growth of LoVo and Caco-2, two human colon tumor cell lines [87]. 10*Z*-Hymenialdisine is now extensively used in research programs and is readily available from biochemical product suppliers as it shows good efficacy *in vivo* and has significant potential in a variety of different disease types as discussed earlier. Also extracted from the same sponge species was hymenin (**59**, Figure 5), which also showed inhibitory activity against the Raf/MEK/MAPK cascade [87]. However, with IC_50_ values ranging from between 128.8 and 250.0 μM for the different specific Raf, MEK and MAPK kinases, hymenin was far less potent than 10*E*-hymenialdisine and 10*Z*-hymenialdisine and was not pursued any further [87].

A methanol fraction of the sponge *Batzella* sp. was found to inhibit Raf kinase with an IC_50_ value of 2.8 μg/mL [88]. The known antimitotic compound halitoxin [89] was identified, however, it was not responsible for the observed kinase inhibitory activity.

Onnamide A (**67**) and theopederin B (**68**) are two compounds that were recently found to induce the stress-activated protein kinases, p38 kinase and JNK [90], two of the subclasses of the MAPK kinase family (Figure 5). While full understanding of the role of JNK in apoptosis has not yet been achieved, it is known that JNK and p38 kinase are predominantly activated by environmental stresses [91]. The JNK pathway is critical in the regulation of apoptosis during early brain development in mice and the p38 MAPK pathway plays a vital role in the production of inflammatory cytokines and subsequent signaling and also appears to be heavily associated with cell survival and proliferation [91]. Onnamide A and theopederin B, heterocyclic compounds that are members of the pederin family isolated from a marine sponge, activate a ribotoxic stress response and induce apoptosis [90,92,93]. As well as inducing the production of p38 and JNK, these two compounds were also found to stimulate plasminogen activator inhibitor-1 (PAI-1) gene expression in concentration ranges of 10–100 nM for onnamide A and 1–10 nM for theopederin B [90]. PAI-1 is an important current drug target as high levels of PAI-1 have consistently been found in human cancer cells and PAI-1 has also been associated with tumor growth, invasion and metastasis [94]. Thus, onnamide A and theopederin B will provide important tools in understanding more about PAI-1 expression and the induction of the ribotoxic stress response [90]. (+)-Makassaric acid (**69**) and (+)-subersic acid (**70**) are novel meroterpenoid compounds, isolated from the sponge *Acanthodendrilla* sp. collected in Indonesia. These compounds were found to inhibit MAPKAP kinase 2 which is involved in stress and inflammatory responses [95].

## 8. Glycogen Synthase Kinase-3 (GSK-3, EC 2.7.11.26)

A serine/threonine protein kinase, the main function of glycogen synthase kinase-3 (GSK-3) is the mediation of glycogen synthase but it is also involved in several key cellular events such as the response to damaged DNA and the phosphorylation of the microtubule associated mammalian protein tau. Overactivity of this phosphorylation has been identified as one of the first events in the onset of neurodegenerative diseases such as Alzheimer’s disease [96]. Over the last two decades, interest in GSK-3 has exponentially increased as its potential as a drug target in many non-curable diseases such as type-2 diabetes, stroke, Alzheimer’s disease, and bipolar disorder is recognized [96]. Current small molecule inhibitors of GSK-3 include pyridyloxadiazoles, thiadiazolidindiones, pyrazolopyrimidines and maleimides [96], but marine sponges are also proving to be a reliable source of secondary metabolites showing inhibitory activity against this drug target.

**Figure 6:**
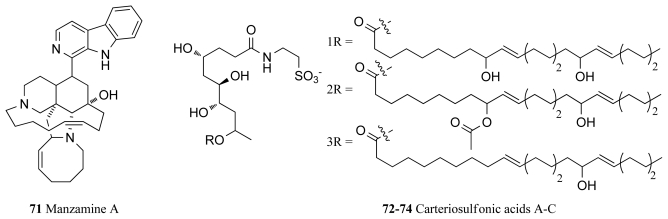
Glycogen synthase kinase-3 inhibitors isolated from marine sponges.

Manzamine A (**71**, Figure 6), a complex alkaloid isolated from an Okinawan sponge of the genus *Haliclona*, is one such compound showing specific non-competitive inhibition of ATP binding in GSK-3β with an IC_50_ value of 10.2 μM [96]. Manzamine A also inhibits CDK-5 with an IC_50_ value of 1.5 μM, and as this kinase coupled with GSK-3 represents the two main players in the hyperphosphorylation mechanism in Alzheimer’s disease, manzamine A is a useful drug lead for the future treatment of this disease [96]. This conclusion is supported by the fact that manzamine A has proved capable of entering cells and interfering with the tau protein as well as causing arrest in the hyperphosphorylation in human neuroblastoma cell lines [96]. Structure-activity relationships between manzamine A and the GSK-3 pharmacophore have been carried out and a variety of manzamine A analogues have also been synthesized indicating that the entire manzamine molecule is required for GSK-3 inhibitory activity [96]. Manzamine A and its synthesized derivative (−)-8-hydroxymanzamine A, have also been identified as promising new antimalarial agents producing *in vivo* inhibition of the growth of the malaria parasite *Plasmodium berghei* in rodents [97]. As the malaria parasite rapidly achieves resistance to currently administered antimalaria drugs, patents for the use of manzamine A in human antimalarial drugs have been submitted [98].

The carteriosulfonic acids A–C (**72**–**74**, Figure 6), novel compounds containing a 4,6,7,9-tetrahydroxylated decanoic acid subunit, were recently identified during a screen to identify modulators of Wnt signaling, which plays a key role in cell proliferation [99]. Phosphorylation of β-catenin by GSK-3β is involved in the negative regulation of Wnt signaling and thus it was proposed that inhibitors of GSK-3β may be associated with Wnt signaling activation. Accordingly, the compounds (**72**–**74**) were isolated from an extract of the marine sponge *Carteriospongia* sp., which was a Wnt signaling activator and were found to be low micromolar inhibitors of GSK-3β. Although further biological studies were foreshadowed in the above article, they had not yet appeared at the time of writing this review [99].

## 9. Other Kinases

**Figure 7:**
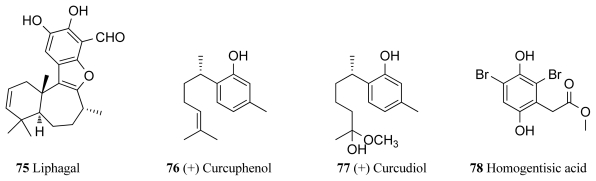
Inhibitors of PI3K, Src, and focal adhesion kinase isolated from marine sponges.

Liphagal (**75**, Figure 7), a meroterpenoid isolated from the marine sponge *Aka coralliphaga* collected in Dominica, was found to exhibit inhibitory activity against PI3K (phosphoinositide-3-kinase) with an IC_50_ value of 100 nM, with 10 folder higher potency against PI3Kα than towards PI3K*γ* [100,101]. This compound also exhibited cytotoxicity against human colon (IC_50_ 0.58 μM) and human breast (1.58 μM) tumor cell lines [100]. This sponge species is also known to produce the PKC inhibitors corallidictyals A and B (**26** and **27**) (see Section 3). Two bisabolene type sesquiterpenoids, (+)-curcuphenol (**76**, Figure 7) and (+)-curcudiol (**77**, Figure 7) were identified as bioactive compounds from the sponge *Axynissa* sp. from Indonesia. Curcuphenol showed Src protein kinase inhibition with an IC_50_ value of 7.8 μg/mL, while curcudiol inhibited focal adhesion kinase (FAK) with an IC_50_ value of 9.2 μg/mL [102]. Protein kinase A inhibitory activities of up to 100% (at 100 μg/mL) along with haemolytic and brine shrimp activities were also observed in a range of extracts isolated from three deep-water sponges collected from North Western Australia [103]. A novel compound, homogentisic acid (**78**) was isolated from the sponge *Pseudoceratina* collected in Vanuatu [104]. The authors previously isolated xestoquinone from a *Xestospongia* sp. collected from the same place and in their research for new antimalarial drugs found that this compound was an inhibitor of Pfnek-1, which is a NIMA-related protein kinase of *Plasmodium falciparum*. Therefore homogentisic acid was also screened against Pfnek-1 and found to display an IC_50_ value of 1.8 μM against this target [104] Hymenialdisine (**4**) has also showed Polo-Like kinase-1 inhibitory activity of 10 μM. It was isolated along with debromohymenialdisine (**5**) and four novel dihydrohymenialdisine derivatives from the sponge *Cymbastela cantharella* [105].

There are several aspects to consider regarding kinase inhibitors such as whether they are ATP-competitive or non-competitive inhibitors and whether the compounds inhibit their reported enzymatic targets in cellular assays. However, the level of mechanistic detail and characterization of the kinase inhibitory activity of the compounds described herein varies greatly. Thus, herein those articles providing a higher degree of characterization are indicated in the Table 1 by an asterisk. A further issue is one of broader kinase selectivity profiling that would be useful to see addressed in the literature, both in terms selectivity of the inhibitors towards other kinases and towards other targets.

## 10. Conclusions

The search for kinase inhibitors from marine sources has proven extremely successful with the advent of compounds such as bryostatin-1 into pharmaceutical development, and others such as hymenialdisine (**4**) and manzamine (**71**) looking promising. In particular, marine sponges are a rich source of highly diverse chemical compounds including lipids, terpenes and alkaloids, enhanced by a high incidence of novel carbon skeletons, such as that of xestocyclamine A (**1**). Marine sponge metabolites have proven to be extremely potent against a range of kinase targets heavily involved in an increasing list of disease mechanisms including cancer, Alzheimer’s disease and atherosclerosis. Several kinase inhibitors such as fascaplysin (**30**) possess strong selectivity not only for specific kinase subtypes, but also for cancerous cells over healthy cells and are thus promising molecules in the development of new oncological pharmaceuticals. With new technological developments bringing access to previously unexplored marine environments such as the deep sea [106], it is certain that many more sponge metabolites with novel structures and potent kinase inhibitory activities will be discovered in the future. Furthermore, as our understanding of the mechanism and regulation of various kinases continues to grow, marine sponge-derived kinase inhibitors are destined to play an expanding role in the treatment of various diseases.

## Figures and Tables

**Table 1:** Various kinase inhibitors isolated from marine sponges.

Kinase	Sponge species	Natural product (or *compound type*)	IC_50_ (μM) ₤ (or *% inhibition*)	Ref.
**PKC**	*Xestospongia* sp.	Xestocyclamine A (**1**)	10	[19]
	*Stylotella aurantium*	Axinohydantoins (**2**, **3**)	9–22	[22]
	*Stylotella aurantium*	Hymenialdisines (**4**, **5**)	0.8–1.3	[22]
	*Dysidea frondosa*	Frondosins A–E (**6**–**10**)	2–31	[23]
	Class *Calcarea*	BRS 1 (**11**)	98	[28] *
	Family *Spongiidae*	Nakijiquinones A–D, G–I (**12**–**18**)	23–270	[29,30]
	*Spongia* sp.	Spongianolides A–E (**19**–**23**)	20–30	[36]
	*Forecpia* sp.	Lasonolide A (**24**)	0.03	[38,40] *
	*Penares sollesi*	Penazetidine A (**25**)	1	[41]
	*Aka coralliphaga*	Corallidictyals A and B (**26**, **27**)	28	[42]
**CDK**	*Axinella verrucosa*	Hymenialdisine (**4**)	0.02	[48] *
	*Microxina* sp.	Microxine (**28)**	13	[52]
	*Kirkpatrickia* v*arialosa*	Variolin B (**29**)	0.03	[53] *
	*Fascaplysinopsis* sp.	Fascaplysin (**30**)	0.4	[61] *
	*Hymeniacidon* sp.	Konbu’acidin A (**31**)	27	[62]
	*Aka* sp.	Quinol derivative (**34**)	0.019	[63]
	*Aka* sp.	Halistanol sulfate (**37**)	0.013	[63]
**TPK**	*Ircinia* sp.	*Prenylhydroquinone 4-sulfates* (**38**–**40**)	7–15	[66]
	*Dysidea* sp.	Melemeleone B (**41**)	28	[68]
	*Xestospongia* sp.	Halenoquinone (**42**, **43**)	1.5–5	[69,73] *
	*Xestospongia* sp.	Halenaquinols (**44**, **45**)	0.6–60	[69] *
	*Xestospongia* sp.	Xestoquinone (**46**)	28	[69] *
	*Xestospongia* sp.	Xestoquinolide A (**47**)	80	[73] *
**EGFR**	*Hymeniacidon* sp.	Tauroacidins A–B (**48**, **49**)	38–45	[75]
	*Suberea* sp.	Ma’edamine A (**50**)	11	[76]
	*Hymeniacidon* sp.	Spongiacidins A–B (**51**, **52**)	19–21	[78]
	*Verongia aerophoba*	Aeroplysinin-1 (**53**)	0.5	[79]
	*Acanthella cavernosa*	*Dibenzofurandiols* (**54**–**57)**	*33–59*†	[81]
	*Spongionella* sp.	3′-Norspongiolactone (**58**)	*25*†	[82]
	*Spongionella* sp.	Gracilins J–L (**59**–**61**)	*19–75*†	[82]
**MAPK**	*Ircinia* sp.	Cheilanthene sesterpenoids (**62**–**65**)	4–90	[84]
**Raf/MAP**	*Stylissa massa*	Hymenialdisines (**4**, **5**)	0.003–0.006	[87] *
	*Stylotella aurantium*	Hymenin (**66**)	129	[87] *
	*Theonella* sp.	Theopederin B (**68**)	- ‡	[90,92] *
	*Theonella* sp.	Onnamide A (**67**)	- ‡	[90,93] *
	*Acanthodendrilla* sp.	(+)-Makassaric acid (**69**)	20	[95]
	*Acanthodendrilla* sp.	(+)-Subersic acid (**70**)	9.6	[95]
**GSK-3**	*Haliclona* sp.	Manzamine A (**71**)	10	[96] *
	Unidentified sp.	*Glycerol lipids* (**72**–**74**)	0.1–0.4	[99]
**Others**	*Aka coralliphaga*	Liphagal (**75**)	0.1	[100,101]
	*Axynissa* sp.	(+)-Curcuphenol (**76**)	36	[102]
	*Axynissa* sp.	(+)-Curcudiol (**77**)	37	[102]
	*Pseudoceratina* sp.	Homogentisic acid (**78**)	1.8	[104]

₤ Values reported in μg/mL were converted to μM;

† % Inhibition at 100 μM;

‡ Induces activation of p38 and JNK;

* An asterisk denotes articles containing detailed characterisation of the kinase inhibitory activity.
